# Muscle strength in adolescent men and risk of cardiovascular disease events and mortality in middle age: a prospective cohort study

**DOI:** 10.1186/1741-7015-12-62

**Published:** 2014-04-14

**Authors:** Simon Timpka, Ingemar F Petersson, Caddie Zhou, Martin Englund

**Affiliations:** 1 Orthopedics, Clinical Sciences Lund, Lund University, Lund, Sweden; 2 Clinical Epidemiology Research & Training Unit, Boston University School of Medicine, Boston, MA, USA

**Keywords:** Cardiovascular disease, Coronary heart disease, Stroke, Muscle strength, Prevention, Epidemiology

## Abstract

**Background:**

Ischemic heart disease and stroke are two severe types of cardiovascular disease (CVD), a major contributor to the global burden of disease. The preventive framework currently includes promotion of both adequate cardiorespiratory and muscular fitness. Although muscle fitness is established as an indicator of health, it is currently unknown whether muscle strength is associated with later CVD independently of cardiorespiratory fitness.

**Methods:**

We studied 38,588 Swedish men who in 1969 to 1970 (typically aged 18 years) completed compulsory conscription. Using the mean standardized score of three isometric muscle strength tests performed at conscription (hand grip, elbow flexion and knee extension), we categorized the subjects into three groups with the 25th to 75th percentile defining the reference category. We followed the cohort until 2012 for diagnosed CVD events and mortality via national health care registers and the national cause of death register. To estimate hazard ratios (HR) for CVD events (coronary heart disease or stroke) and CVD mortality we used Cox proportional hazard models adjusted for body mass index, smoking, alcohol consumption, cardiorespiratory fitness and socioeconomic status.

**Results:**

Men with high muscle strength in adolescence had a decreased risk of later CVD events (HR 0.88, 95% confidence interval 0.77 to 0.99), whereas we observed no increased risk in men with low muscle strength (0.99, 0.86 to 1.13). However, low muscle strength was associated with increased risk of CVD mortality during middle age (1.31, 1.02 to 1.67).

**Conclusions:**

Muscle strength in adolescent men is inversely associated with later CVD events and CVD mortality in middle age, independently of cardiorespiratory fitness and other important confounders. Thus, the role of muscle fitness in the prevention and pathogenesis of CVD warrants increased attention.

## Background

Ischemic heart disease and stroke are major contributors to the global burden of disease [1]. Pleasingly, the last decades we have experienced major advances in both pharmacological and invasive treatments, which have greatly improved the prognosis for patients after a major cardiovascular disease (CVD) event. However, primary and secondary prevention are still important, both to decrease the individual suffering as well as to decrease the societal burden. Important cornerstones of the preventive framework include a balanced diet, no smoking and adequate levels of physical activity [2]. Increasing physical activity generally increases physical capacity, with muscular function and cardiorespiratory fitness (CRF) being its two main components. CRF is a well-established predictor of future CVD morbidity [3], influencing the development of disease as well as increasing the physiological margin if a CVD event occurs. With low muscle fitness being an established predictor of mortality and low health in older populations [4,5], there is also increasing epidemiological evidence that muscle strength is associated with all-cause mortality independently of CRF in adults [6-8]. In addition, there are previous studies suggesting an association between muscle strength and CVD risk factors, such as arterial stiffness [9] and long term weight gain [10]. Currently, the World Health Organization recommends adults to perform muscle strengthening activities on at least two separate days per week [11]. Muscle strengthening activities might potentially be a better alternative for people who are not motivated to, or due to medical reasons cannot, participate in more focused cardiorespiratory exercise. However, it is currently unknown whether muscle strength is associated with later CVD independently of CRF [12] although a crude association has been reported [13].

Hence, our primary aim was to investigate the association between overall isometric muscle strength in late adolescence [14] and later CVD events in men, independently of CRF. As a secondary aim, we assessed the risk of CVD mortality. We hypothesized that overall isometric muscle strength in late adolescence is associated, not necessarily linearly, with subsequent CVD events and CVD mortality. With follow-up until late middle age (up to 40 years of follow-up) and careful consideration of key confounders, we have addressed important limitations of previous studies.

## Methods

**Figure 1:**
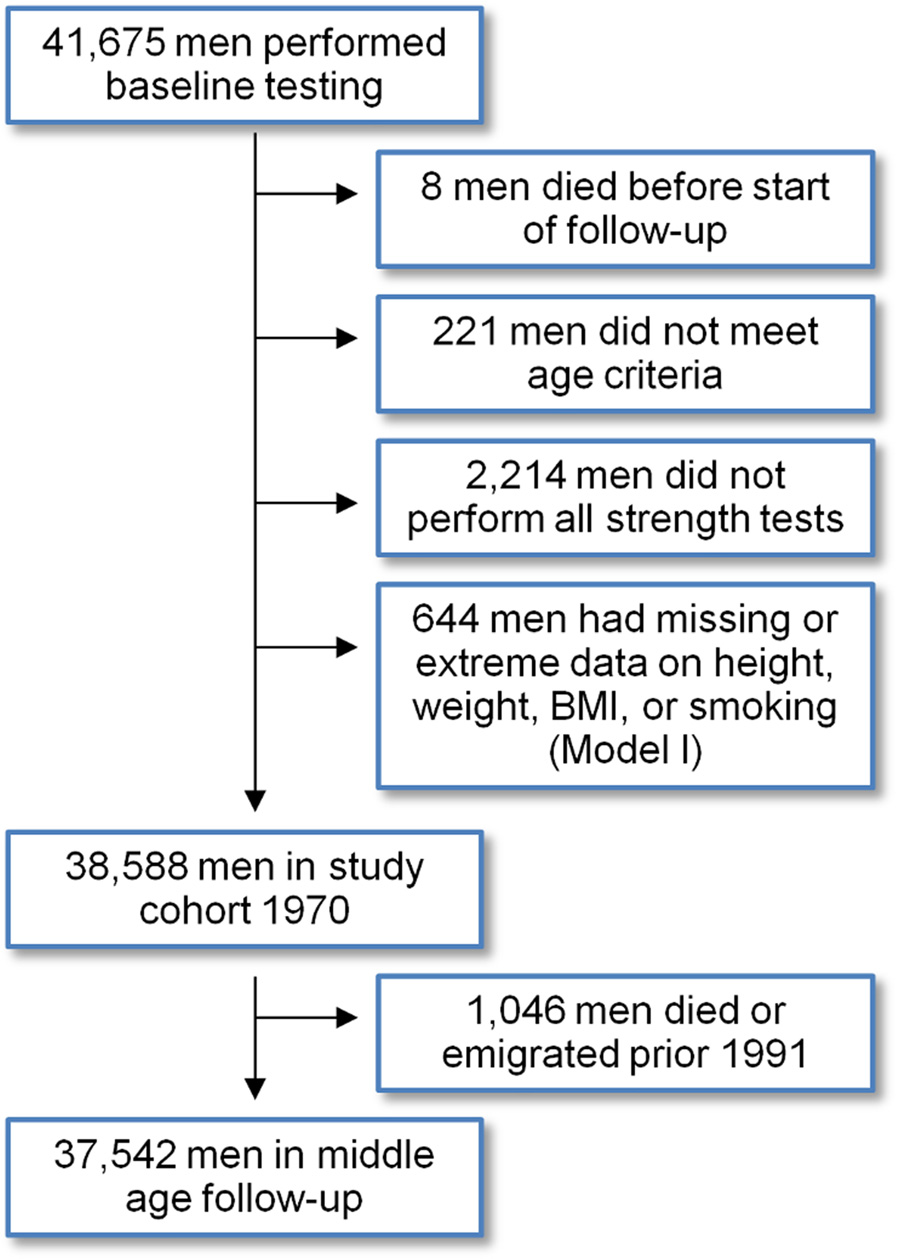
The identification of the study sample and the loss during follow-up.

For this prospective register-based cohort study, we identified 38,588 Swedish men (Table 1). Typically aged 18 years, the men were tested and examined during conscription for military service between September 1969 and May 1970 (Figure 1). In 2012, we collected data on the cohort regarding CVD events and mortality until late middle age via Swedish registers on in-patient care and cause of death. The study was approved by the Regional Ethical Review Board at Lund University (2011/500) and is reported according to the STROBE (STrengthening the Reporting of OBservational studies in Epidemiology) guidelines [15].

**Table 1:** Descriptive statistics of total study sample and the categories of muscle strength

		**Categories of muscle strength**		
**Characteristics**	**Total sample**	**Low**	**Average**	**High**
	**n = 38,588**	**n = 9,647**	**n = 19,294**	**n = 9,647**
Mean age at start of follow-up	19.0	19.0	19.0	19.0
Mean height in centimeters (SD)	178.0 (6.3)	176.4 (6.5)	178.0 (6.2)	179.5 (6.1)
Mean mass in kilograms (SD)	66.5 (9.3)	60.9 (7.8)	66.3 (8.0)	72.4 (9.4)
Mean arm flexion strength in N (SD)	370 (79)	292 (39)	364 (48)	460 (69)
Mean knee extension strength in N (SD)	533 (103)	431 (67)	532 (69)	638 (85)
Mean hand grip strength in N (SD)	608 (98)	508 (63)	605 (64)	711 (77)
Body mass index (%)				
<18.5	5,323 (13.8)	3,169 (32.8)	1,971 (10.2)	183 (1.9)
18.5 to 24.9	30,697 (79.6)	6,246 (64.7)	16,340 (84.7)	8,111 (84.1)
≥25 to 29.9	2,242 (5.8)	204 (2.1)	880 (4.6)	1,158 (12.0)
≥30	326 (0.8)	28 (0.3)	103 (0.5)	195 (2.0)
Smoking (%)				
Non-smoker	15,754 (40.8)	4,125 (42.8)	7,664 (39.7)	3,965 (41.1)
1 to 10 daily cigarettes	12,646 (32.8)	3,128 (32.4)	6,438 (33.4)	3,080 (31.9)
10+ daily cigarettes	10,188 (26.4)	2,394 (24.8)	5,192 (26.9)	2,602 (27.0)
Alcohol as grams/week (%)*	n = 37,620	n = 9,401	n = 18,799	n = 9,420
0	2,331 (6.2)	738 (7.9)	1,058 (5.6)	535 (5.7)
1 to 100	22,923 (60.9)	5,643 (60.0)	11,473 (61.0)	5,807 (61.6)
101 to 250	9,529 (25.3)	2,354 (25.0)	4,800 (25.5)	2,375 (25.2)
251+	2,837 (7.5)	666 (7.1)	1,468 (7.8)	703 (7.5)
Cardiorespiratory fitness (%)*	n = 30,941	n = 7,174	n = 15,694	n = 8,073
Low	15,509 (50.1)	3,403 (47.4)	7,910 (50.4)	4,196 (52.0)
High	15,432 (49.9)	3,771 (52.6)	7,784 (49.6)	3,877 (48.0)
Attained education 1990 (%)*	n = 35,332	n = 8,815	n = 17,678	n = 8,839
9 years or less	12,534 (35.5)	2,887 (32.8)	6,057 (34.3)	3,590 (40.6)
Secondary	14,188 (40.2)	3,432 (38.9)	7,270 (41.1)	3,486 (39.4)
Higher	8,610 (24.4)	2,496 (28.3)	4,351 (24.6)	1,763 (19.9)
Parental socioeconomic status (%)*	n = 38,581	n = 9,645	n = 19,290	n = 9,646
Blue collar	19,469 (50.5)	4,674 (48.5)	9,729 (50.4)	5,066 (52.5)
Lower white collar	10,986 (28.5)	2,696 (28.0)	5,492 (28.5)	2,798 (29.0)
Upper white collar	6,214 (16.1)	1,822 (18.9)	3,110 (16.1)	1,282 (13.3)
Other	1,912 (5.0)	453 (4.7)	959 (5.0)	500 (5.2)

SD = Standard deviation.

*The numbers represent the subjects with covariate information available.

Until 2010, conscription was mandatory by law for all Swedish men. In 1969/1970, only persons with serious functional impairment, such as congenital disorders, were exempted. Six regional test offices were used, with each testing young men in its catchment area. During a two-day session, the men performed a battery of physical and mental tests and were also evaluated by a medical doctor and a psychologist.

A small minority of subjects have incongruous anthropometric data registered. Therefore, we excluded men with an extreme value on weight (<40 kg, >150 kg), height (<150, >210 cm) or body mass index (BMI, <15, >60 kg/m^2^). To only include men in late adolescence, we also excluded a small minority of men whom were younger than 17 years or older than 19 years during conscription. To control for censoring during follow-up, we used data on emigration and immigration.

### Muscle strength measurement

As a measure of general muscle strength, we used the mean standardized score of three isometric muscle strength tests; hand grip strength, elbow flexion and knee extension. At baseline, the muscle strength was measured as previously described [16] (personal communication L-O Nordesjö, 19 December 2012). However, the exact test protocol was not available due to military restrictions. In summary, a specially constructed “muscle chair” was used with strain gauges (KRK-2 and KRG-4) and indicator (BKI-1) from AB Bofors modified by AB Medicinska Apparater, Södertälje, Sweden. Hand grip strength was measured with the hand in a vertical position, with 90° flexion at the elbow, and the humerus being parallel with the torso. Elbow flexion and knee extension were measured in a sitting position with 90° flexion over the main joint and with straps over the radial styloid process and 5 cm above the most distal part of the fibular malleolus, respectively. Using percentiles of the mean standardized score (standardized score = (value - mean)/standard deviation), we categorized the cohort into low, average and high strength with the 25th and 75th percentiles as cut offs (that is, the category average strength contained 50% of the sample). By using the mean of the standardized muscle strength scores, the relative strength of each muscle group is equally contributing to the composite muscle strength measure.

### Covariates

Weight was measured to the nearest kilogram. Height was measured to the nearest centimeter. We calculated BMI as weight/height^2^. Smoking at baseline was categorized into three groups (non-smokers, 1 to 10 cigarettes per day and >10 cigarettes per day). Self-reported total intake of alcohol was measured in grams per week as previously reported [17] and categorized into four groups (0, 1 to 100, 101 to 250, >250).

As a measure of CRF, we used physical work capacity (in relation to body weight) categorized as low or high. Subjects who were not limited by disease, including a present cardiovascular condition, performed a maximal test (W_max6_) on a cycle ergometer (Elema-Schönander Model AM 368 or similar) [18,19]. The test has been reported to correlate well with VO_2_max in young male students (r = 0.9) [20,21], though the exact test protocol was not available to us due to military restrictions. In short, each subject started on the same load (230 W) and worked until exhaustion or the prescribed pedaling rate (60 rotations/minute) could not be upheld. For a minority still working at 12 minutes, further increments of 30 W were made every 6 minutes. We excluded subjects who did not reach 90% of age-predicted maximal heart rate (MHR) at the end of testing, that is, <175 beats/minute (MHR = 208 - 0.7 * 18.5) [22]. The test results were registered as stanine scores with known corresponding intervals of physical work capacity in W. A stanine scale is based on the normal distribution and usually used for standardized testing. The scale has nine increments, the first and ninth step each delimit 4% of the population whereas step five constitutes 20% [23]. To allow estimation of physical work capacity in relation to body weight as a proxy for CRF, we estimated the exact result for each conscript using the mean of the upper and lower limit of each stanine score interval. Exceptions were subjects with the highest stanine score, which we categorized as having high fitness, independent of their body weight. Otherwise, those with the highest score weighing the most would be systematically categorized as having low CRF.

We collected data on adult and childhood socioeconomic status from Statistics Sweden. As a proxy for the conditions during childhood, we used information on parental occupation and education in 1960. For a minority of subjects with missing values, the self-reported parental socioeconomic status at conscription was used. For categorization into four groups, we used a white/blue collar approach. For adult socioeconomic status, we used attained level of education in 1990.

### Statistical analyses

All statistical analyses were made in SAS 9.3 (SAS Institute, Cary, NC, USA). We used Cox proportional hazard models to calculate hazard ratios (HR) for outcomes and control potential confounders. In model I, we adjusted for BMI and smoking. In model II, we further adjusted for alcohol, CRF and socioeconomic status. For the middle age analyses, all men in the cohort living in Sweden on 1 January 1991, were included in model I (37,542 men). For model II (main model), the corresponding number was 27,766 men (74.0%). For the mortality analysis starting in 1970, 30,186 men (78.2%) had complete information in model II.

Our primary endpoint was time to CVD event in middle age. Due to the start of data registration on hospitalization and attained education, the actual follow-up regarding ischemic CVD started when the men were typically aged 39 and continued for 20 years through middle age. In other words, for all men alive 1 January 1991, we collected data on hospitalizations with a CVD diagnosis until 31 December 2010 from the nation-wide Swedish in-patient register [24]. With few exceptions, all diagnoses from in-patient care in Sweden are reported to the National Board of Health and Welfare. We defined CVD events as coronary heart disease or stroke morbidity (including hemorrhagic stroke but excluding transient ischemic attack) according to diagnoses based on the International Classification of Disease (ICD) version 9 (410 to 414, 430 to 434, 436 to 438, 342, 344) and 10 (I20 to I25, I60 to I66).

Starting 1 June 1970, we followed up on all deaths until 2 August 2012. However, the cause of death registered according to the ICD (versions 8, 9 or 10) was not available for deaths in 2012. Thus, the analyses of all-cause and specific mortality differed by seven months in time to follow-up. For the period 1970 to 2011, we separately identified deaths due to CVD (ICD-8, ICD-9: 390 to 459 ICD-10: I00 to I99). We also separately investigated CVD and all-cause mortality in middle age (1991 to 2011), the period for which morbidity data was available.

### Sensitivity analyses

To investigate if the measure of socioeconomic status influenced the results, we also investigated socioeconomic status as the socioeconomic index (SEI) 1990. Furthermore, we investigated if year of birth, marital status, height, exclusion due to heart rate <175 at the end of CRF testing, the conscription office or blood pressure (potential mediator) had any influence on HR estimates. Using the first quintile of mean muscle strength as reference, we also tested trend across groups of muscle strength. In separate analyses, we also excluded subjects with CVD (ICD 8: 390 to 459) or psychiatric disease (ICD 8: 290 to 315) at baseline.

## Results

Low muscle strength was associated with low BMI (Table 1). During 710,019 person-years of follow-up in middle age (1991 to 2010), 1,944 men (2.7 per 1,000 person-years) were hospitalized with CVD events. Men with high muscle strength had a statistically significant decreased HR for CVD events in the fully adjusted (main) model (Table 2).

**Table 2:** Hazard ratio of CVD events and mortality by muscle strength with average strength as reference

	**Low strength (HR, 95% CI)**		**High strength (HR, 95% CI)**	
**Outcome**	Model I*	**Model II**^**†**^	Model I*	**Model II**^**†**^
Middle age^‡^ morbidity				
CVD events^**§**^	0.99 (0.88 to 1.10)	0.99 (0.86 to 1.13)	0.91 (0.81 to 1.02)	**0.88** (0.77 to 0.99)
Middle age mortality				
CVD	**1.23** (1.01 to 1.51)	**1.31** (1.02 to 1.67)	1.11 (0.92 to 1.34)	0.98 (0.78 to 1.24)
All-cause	**1.17** (1.06 to 1.29)	**1.18** (1.04 to 1.33)	1.03 (0.94 to 1.14)	0.98 (0.87 to 1.10)
Total^**||**^ mortality				
CVD	**1.28** (1.06 to 1.55)	1.21 (0.96 to 1.51)^**¶**^	1.08 (0.90 to 1.30)	1.03 (0.84 to 1.28)^**¶**^
All-cause	**1.16** (1.07 to 1.27)	**1.14** (1.03 to 1.26)^**¶**^	1.03 (0.95 to 1.12)	1.04 (0.95 to 1.15)^**¶**^

CI, Confidence interval; CVD, Cardiovascular disease; HR, Hazard ratio.

Significant results are in bold (α = 0.05).

* = Adjusted for body mass index, smoking.

^**†**^ = *Main model*. Adjusted for body mass index, smoking, alcohol, cardiorespiratory fitness, education (n = 27,766, that is, 74.0% of subjects in model I).

^**‡**^ = typically from age 39 years (1991 to 2010).

^**§**^ = coronary heart disease or stroke diagnosis (excluding transient ischemic attack) from in-patient care.

^**||**^ = typically from age 19 years (1970 to 2011/2012).

^**¶**^ = Model includes parental socioeconomic status instead of the subject’s education as follow-up starts in adolescence (n = 30,186, that is, 78.2% of subjects in model I).

During 1,569,422 person-years of total follow-up, 3,390 men (2.2 per 1,000 person-years) died with 2,610 (77.0%) deaths occurring in middle age (1991 to 2012). A total of 167 men died in 2012 and had yet to be assigned a cause of death at the time of data collection due to a lag in the registration. Of the 3,223 deaths with a registered cause of death, 687 (21.3%) were due to CVD, of which 629 (91.6%) occurred in middle age. Men with low muscle strength had a statistically significant increased HR of middle age CVD mortality after adjusting for BMI, smoking, alcohol, CRF and attained education. For total mortality, the HRs were moderately increased for both CVD and all-cause but appeared significant in the fully adjusted model only for the latter.

### Sensitivity analyses

Year of birth and conscription office did not alter the pattern of HR estimates. In general, being unmarried was associated with an increased risk of the outcomes, although we observed no major changes in the HR estimates of muscle strength. High diastolic blood pressure was typically associated with having a cardiovascular outcome but had only minor effects on risk estimates. Adjusting for SEI instead of education or including subjects independently of the heart rate level at the end of CRF testing did not affect the overall pattern of association. Adding height to the fully adjusted models, the observations regarding all-cause and CVD mortality for the low strength group remained essentially unchanged, whereas the association with CVD events in the high strength group were slightly attenuated (HR 0.92, 0.80 to 1.04). We observed no trend across muscle strength groups for any of the outcomes. When we excluded men with a CVD (n = 1,271) or a psychiatric diagnosis (n = 4,324) at baseline, we observed only minor changes of HRs and the overall patterns remained.

## Discussion

Our most important observation is that high muscle strength in young men was associated with lower risk of future CVD events, independently of well-known risk factors such as CRF, smoking and BMI. As confirmed by a recent review [12], this is a novel finding. In addition, we also report an increased risk of middle age CVD mortality in men with low muscle strength, independently of common risk factors.

### Muscle strength as a risk factor for cardiovascular disease

With observations that suggest an inverse association between muscle strength in young men and later CVD, the pathways of potential causality remain as a central question. Does high muscle strength add CVD protection apart from CRF or is it merely a proxy for a more favorable future CVD risk profile? Notably, there are previous studies supporting a biological link between muscle strength and later CVD, such as obesity protection [6] through increased resting energy expenditure [25] and lower arterial stiffness in young men with high muscle strength [9]. Early developmental aspects might also play a role as birth size is associated with both low adult muscle strength [26] as well as ischemic heart disease [27]. Whereas we observed a decreased risk of CVD events in men with high muscle strength, the group had no decreased risk of CVD mortality. In contrast, having low muscle strength was associated with increased risk of CVD mortality but not for CVD events. Potentially, this could be explained by muscle strength having different thresholds of protective effect depending on the severity of the outcome measurement. In the context of this study, there is scarce support in the literature that any of the muscle strength tests would be particularly associated with CRF and CVD. Thus, we have not separately investigated each muscle strength test and its association with the outcomes to reduce the number of primary analyses and avoid a data mining approach. We chose to focus the main analyses on detecting non-linear associations as we found it more feasible to use the average muscle strength group as reference from a public health perspective. Furthermore, the known variability in muscle adaption following training would support a non-linear association [28].

Low muscle strength in adolescent men has previously been linked to an increased risk of later suicide mortality [29]. It is plausible that subjects with psychiatric disease or CVD at baseline perform less well on physical capacity test in general, possibly introducing bias. However, when we excluded subjects with CVD or psychiatric disease at baseline, the risk estimates were only marginally altered. When we adjusted for conscription office, a few offices appeared statistically significant without altering the risk estimates substantially.

### Strengths and limitations

The study includes a large population-based sample, includes prospectively ascertained exposure information and baseline data on known confounders, has a long follow-up of four decades, and small loss during follow-up using comprehensive registers. However, there are also limitations that should be considered when interpreting the results. First of all, we do not know how the motivation for military service may have biased our observations. However, as few men were fully discharged, most hypothetical underachievers would still have to enlist, possibly raising motivation for testing. Although morbidity data for CVD events are missing in young adulthood, the incidence of coronary heart disease and ischemic stroke is low prior to middle age [30,31]. Thus, the large majority of cases should still have been identified with the present study design. Also, if our hypothesis is correct, early cases not registered would at most dilute our results. It should also be noted that all conscripts included in the study did not perform a maximum work capacity test. As non-performers typically have lower muscle strength than performers, this should also at most dilute our results. Furthermore, CRF was not directly measured but approximated from a physical work capacity test on a bicycle. Compared to the treadmill testing used in other studies in the area [6,8], the CRF variable might be slightly inferior. However, CRF had a statistically significant effect on the HR estimate of total CVD mortality (low vs. high 1.33, 1.10 to 1.60). Noticeably, the study design also allows us to adjust the risk estimates for the mediating effects of attained education. Previous studies have reported that changes of behavior or physical fitness during adulthood affect the mortality risk [32,33], data not available for the cohort. However, when we adjust for the attained education of subjects in their late 30s, we take some of the environmental differences in early adulthood into account. Although widely used in both primary and secondary screening of CVD, we do not have any data on biochemical markers of CVD risk, such as cholesterol levels. However, we first and foremost regard the metabolic profile of the individual as an intermediate variable between fitness and later CVD and not as a confounder. We have not been able to study the effects of physical activity level on our observations as these data were not available for the cohort. Thus, physical activity level might mediate unmeasured confounder effects. From a statistical viewpoint, it is important to note that Cox proportional hazard models might produce biased results when studying other outcomes than all-cause mortality due to competing risks (that is, death) [34]. However, we did not observe any major differences between subgroup curves of cumulative incidence compared to the corresponding curves adjusted for competing risks.

### Generalization and future perspectives

In brief, as long as the limitations of the study are acknowledged, we find our observations generalizable to a male population of European ancestry. The study does not include women as only men were subjected to conscription testing. Furthermore, as the muscle mass and isometric muscle strength greatly differ among young males and females, our study does not give sufficient evidence to suggest a similar association in women as in men. Although the source population was ethnically homogenous and predominantly white, muscle strength has been shown to be an important predictor of mortality also in other ethnicities, such as men of Japanese ancestry [35].

We consider muscle fiber type distribution (type I or type II) to be an area of interest for future research. Muscle fiber type distribution has a major congenital component [36], is affected by physical activity [37], and is associated with isometric muscle strength [38] as well as obesity and type 2 diabetes [39,40]. With promising techniques that measure muscle fiber type non-invasively [41], large cohort studies on how muscle fiber type distribution is associated with CVD are possible. It is also important to better understand how fitness interventions alter the CVD risk for individuals with different levels of baseline fitness.

## Conclusions

This study provides novel evidence that overall isometric muscle strength in young men is inversely associated with later risk of CVD events as well as CVD mortality in middle age, independently of CRF. We suggest that the role of muscular function in the pathogenesis of CVD, and the histological and biochemical links that hypothetically might explain our observations, warrant increased attention and further investigation.

## Abbreviations

BMI: Body mass index; CI: Confidence interval; CRF: Cardiorespiratory fitness; CVD: Cardiovascular disease; HR: Hazard ratio; ICD: International classification of disease; MHR: Maximum heart rate; WHO: World Health Organization.

## Competing interests

All authors declare that there are no conflicts of interest.

## Authors’ contributions

ST provided the hypothesis, collected the data, contributed to data analysis and drafted the manuscript. ST, IFP, CZ and ME planned the study. CZ analyzed the data. ME, IFP and CZ critically revised the manuscript. All authors approved the final version to be published.

## Pre-publication history

The pre-publication history for this paper can be accessed here:

http://www.biomedcentral.com/1741-7015/12/62/prepub
